# Regional Variation across Canadian Centers in Radioiodine Administration for Thyroid Remnant Ablation in Well-Differentiated Thyroid Cancer Diagnosed in 2000–2010

**DOI:** 10.1155/2016/2867916

**Published:** 2016-11-29

**Authors:** I. Rachinsky, M. Rajaraman, W. D. Leslie, A. Zahedi, C. Jefford, A. McGibbon, J. E. M. Young, K. A. Pathak, M. Badreddine, S. De Brabandere, H. Fong, S. Van Uum

**Affiliations:** ^1^Department of Nuclear Medicine, Western University, London, ON, Canada; ^2^Department of Radiation Oncology, Dalhousie University, Halifax, NS, Canada; ^3^Department of Internal Medicine, University of Manitoba, Winnipeg, MB, Canada; ^4^Department of Endocrinology, Women's College Hospital, Toronto, ON, Canada; ^5^Department of Radiology and Nuclear Medicine, Memorial University, St. John's, NL, Canada; ^6^Department of Medicine, Dalhousie University, Halifax, NS, Canada; ^7^Department of Surgery, McMaster University, Hamilton, ON, Canada; ^8^Department of Surgery, University of Manitoba, Winnipeg, MB, Canada; ^9^Department of Medicine, Western University, London, ON, Canada

## Abstract

*Background*. Use of radioactive iodine (RAI) ablation has been reported to vary significantly between studies. We explored variation in RAI ablation care patterns between seven thyroid cancer treatment centers in Canada.* Methods*. The Canadian Collaborative Network for Cancer of the Thyroid (CANNECT) is a collaborative registry to describe and analyze patterns of care for thyroid cancer. We analyzed data from seven participating centers on RAI ablation in patients diagnosed with well-differentiated (papillary and follicular) thyroid cancer between 2000 and 2010. We compared RAI ablation protocols including indications (based on TNM staging), preparation protocols, and administered dose. We excluded patients with known distant metastases at time of RAI ablation.* Results*. We included 3072 patients. There were no significant differences in TNM stage over time. RAI use increased in earlier years and then declined. The fraction of patients receiving RAI varied significantly between centers, ranging between 20–85% for T1, 44–100% for T2, 58–100% for T3, and 59–100% for T4. There were significant differences in the RAI doses between centers. Finally, there was major variation in the use of thyroid hormone withdrawal or rhTSH for preparation of RAI ablation.* Conclusion*. Our study identified significant variation in use of RAI for ablation in patients with well-differentiated thyroid cancer both between Canadian centers and over time.

## 1. Introduction

The incidence of thyroid cancer has been rising steadily over the last several decades. Davies and Welch reported a 2.4-fold increase in incidence of thyroid cancer from 1973 to 2002 in their study using the SEER database which contains population-based data on cancers from the USA [1]. Statistics Canada also reported an increase in thyroid cancer prevalence in Canada; prevalence proportion of thyroid cancer was observed to have an average annual increase of 7.4% from 1994 to 2008, the second highest among all cancers reported [2].

The presentation and treatment of thyroid cancer have evolved considerably over the last two decades. The indications for radioactive iodine (RAI) ablation, the administered dose, and administration protocol (thyroid hormone withdrawal versus stimulation by recombinant human TSH (rhTSH)) have changed over time, partly prompted by two studies comparing various RAI 13I activities for remnant ablation [3, 4], prompting updates in the American Thyroid Association (ATA) guidelines [5].

Haymart et al. [6] studied care for thyroid cancer patients in the USA and found considerable regional variation that persisted after controlling for patient and hospital characteristics. Canada and the USA are ethnically and culturally similar. Canada provides health care through a single-payer, government-funded system, with the provinces carrying the primary responsibility for delivering health care. The Canadian health care system aims to provide universal coverage for all medically necessary health care services, so that individuals receive the care they need based on their disease rather than other factors such as socioeconomic status and area of residence.

Currently, information on variation in thyroid cancer care across Canada is limited. A written survey in 2006 suggested significant regional differences in radioactive iodine remnant ablation in both Canada and the USA [7]. We created a collaborative group, Canadian Network for Cancer of the Thyroid (CANNECT), to describe and analyze the care of thyroid cancer within the Canadian context. For the present study, we focused on regional variation in initial RAI ablation across participating Canadian centers and changes over time for the period of 2000 to 2010.

## 2. Methods

### 2.1. Patients/Participating Centers

Patients were recruited from the thyroid cancer clinics at seven Canadian centers that participate in CANNECT and that had each enrolled at least 30 patients. The centers include London, Ontario (ON); Hamilton, ON; Toronto, ON; Winnipeg, Manitoba (MB); Halifax, Nova Scotia (NS); Fredericton, New Brunswick (NB); and St. John's, Newfoundland and Labrador (NL). All centers are regional referral centers, with exception for CancerCare Manitoba which collects information from all thyroid cancer patients within the entire province, with treatment focused within two locations. Some centers (London, Winnipeg, St. John's, and Toronto) have data available starting in 2000, while other centers started to collect data in later years (2006 and onwards). For the present study, patients were included if they had undergone thyroidectomy for well-differentiated papillary or follicular thyroid cancer, they were diagnosed at age 18 or older, and information on RAI ablation was available; patients with known distant metastatic disease at presentation were excluded. Patients who received radiation therapy for locally invasive cancer were not excluded.

Each participating center received study approval by the research ethics boards at their respective institutions. For patients in Manitoba, data collection is mandatory and occurred through the CancerCare Manitoba registry. Access to data is subject to protocol review and approval.

### 2.2. Data Collection

All centers collected core data elements related to thyroid cancer diagnosis and treatment. The core data elements collected related to presentation of thyroid cancer, including year of diagnosis, age at diagnosis, sex, initial surgical management, pathology, staging, and RAI administration.

Individual cases of thyroid cancer were staged according to the American Joint Committee on Cancer (AJCC) tumor-node-metastasis (TNM) classification system (7th edition, 2009). All patients were retrospectively reclassified based on the clinical information collected in the database. Information pertaining to clinical and pathological stage of thyroid cancer at presentation was obtained from clinic notes prepared by treating physicians and cross-referenced with original pathology and/or imaging reports. All data was deidentified to protect patients' privacy and confidentiality and submitted for central data analysis. The data has been carefully verified, and we checked for potential duplicates to avoid including the same patient more than once.

### 2.3. Radioactive Iodine Ablation

We asked all centers to describe how they would prepare for RAI ablation and if any changes in their practice had occurred between 2000 and 2010. We analyzed the RAI ablation in relation to primary tumor size, presence of invasive growth, and presence or absence of nodal neck disease at presentation. For analysis of the administered RAI activities, we created five categories for activities of <1.45 GBq, 1.45–<2.75 GBq, 2.75–<4.6 GBq, 4.6–<6.5 GBq, and ≥6.5 GBq.

### 2.4. Data Analysis

Descriptive data are presented as mean ± SD or percentage as appropriate. We describe data in two ways. First, we compare the participating centers with respect to RAI ablation. Secondly, we describe trends over time for aggregate data for all centers and describe parameters specified per calendar year. Trends over time (2000–2010) were analyzed using the Cochran-Armitage test. Only some centers (London, Winnipeg, St. John's, and Toronto) included patients before 2006; therefore, a separate trend analysis was performed for 2006–2010. Chi square tests were used to compare fractions, and statistical significance was accepted at *P* value less than 0.05.

## 3. Results

### 3.1. Patients and Participating Centers

Overall, 3357 patients with thyroid cancer diagnosed between 2000 and 2010 were identified by the seven centers; 285 patients were excluded because the TNM stage could not be determined, histology information was incomplete, they had distant metastatic disease, or they had pathology other than papillary or follicular DTC (such as medullary, anaplastic, or poorly differentiated).

The final study population included 3072 patients from seven centers. Baseline characteristics for all patients from all centers combined are presented in Table 1. Papillary thyroid cancer was found in 2874 patients, and follicular thyroid cancer in 198 patients. The number of patients enrolled per year increased from 2000 to 2007, after which it appeared to stabilize (Table 2). Based on the lymph node status, we subdivided the patients into two groups: a group with known nodal involvement (N1a/N1b) and a group with no nodal disease or nodal status unknown (N0/Nx). Specified per T stage, the percentage of patients with N1a/N1b was 14% for T1, 14% for T2, 35% for T3, and 70% for T4. For T1, the distribution remained stable over time. For T2 and T3 tumors, the fraction of N1a/N1b positive tumors increased from 2000 to 2010 (*P* = 0.03 and *P* < 0.01, resp.) but did not change from 2006 to 2010. For T2 and T4 tumors, the fraction of N0/Nx decreased over time from 2000 to 2010 (*P* < 0.01 for both). For the 2006–2010 period, such a decrease was found for T2 but not for T4 tumors.

The total number of patients per center, specified per stage, and lymph node status is shown in Table 3. The number of patients per center varied from 1264 in London to 38 in Fredericton. There was no major difference in distribution of lymph node status per stage between centers.

### 3.2. Trends in RAI Administration over Time

The fraction of patients receiving RAI, specified per stage, varied considerably between 2000 and 2010 (Table 4). Overall, there was an increase in the percentage of patients receiving RAI between 2000 and 2005, after which there was a decrease that had not yet stabilized in 2010. For T1 tumors, patients with documented lymph node involvement were more likely to receive RAI than patients with no known lymph node involvement (*P* < 0.01), while, for T2, T3 and T4 tumors, the fraction of patients receiving RAI did not differ between the lymph node positive and lymph node negative group (*P* = NS for all).

The time trend for the RAI dose specified per T stage (T1, T2, and T3) varied depending on lymph node status (data not shown). Overall, for patients with negative/unknown (N0/Nx) lymph node involvement, there was little dose variation between 2000 and 2010. In contrast, for patients with lymph node involvement (N1a/N1b), there was considerable variation in administered RAI activity over time. For T1, T2, and T3, there were an increase in the percentage of patients receiving 5.5 GBq and a decrease in the percentage of patients receiving 3.7 GBq. Most of the changes occurred between 2004 and 2006.

### 3.3. RAI Ablation Protocols for All Centers

Information on indications and administered doses for initial RAI ablation was obtained from seven centers and is presented in Table 5(a). Five centers used guidelines based on locally derived protocols, with the CancerCare Manitoba guidelines being very similar to the ATA guidelines. Two centers stated they used the ATA guidelines. Most centers gave increasing RAI doses for patients with higher TNM stages. Two centers (Toronto and Hamilton) indicated the RAI doses had decreased over the period from 2000 to 2010; other centers did not indicate any changes. Several centers (London, Halifax, and Toronto) stated they had become more selective in the indication for RAI ablation over this time period.

Information on the specific preparation used for RAI ablations is presented in Table 5(b). All centers used a low iodine diet for about 9–14 days. One center (Manitoba) always used thyroid hormone withdrawal (THW) protocol and another center (London) used rhTSH for most patients treated since 2000. When THW was used, the withdrawal period varied from 3 to 6 weeks. Liothyronine was used by 3 centers, with the dose varying between 50 and 75 microgram daily, and was discontinued two weeks before RAI ablation.

### 3.4. RAI Ablation Variation across Participating Centers

Overall, the percentage of patients receiving RAI ablation varied considerably between centers, from 40% in Manitoba to 91% in Newfoundland (Table 6). The fraction of patients receiving RAI was usually higher for patients with lymph node involvement, particularly for T1 and T2 disease, and increased with stage. There was considerable variation between centers. For patients with lymph node involvement, the percentage of patients receiving RAI varied from 50 to 96% for T1, from 73 to 100% for T2, from 57 to 100% for T3, and from 83 to 100% for T4. For patients with negative or unknown lymph node status (N0/Nx), the percentage of patients receiving RAI varied from 15 to 83% for T1, from 33 to 96% for T2, from 54 to 97% for T3, and from 89 to 100% for T4.

Next, we analyzed RAI dose distribution for each center specified for T stage. There was significant variation between centers. One center (Manitoba) gave an activity of ≤1.8 GBq in almost half (48%) of patients; this low dose was used less frequently in the other centers, which predominantly used activities of ≥3.7 GBq. One center (Toronto) used an activity of 3.7 GBq for a large majority of patients. Three centers (London, Halifax, and Fredericton) mainly used either 3.7 GBq or 5.5 GBq. Other centers, such as St John's, Manitoba, and Hamilton, used a wider dose range.

### 3.5. Thyroid Hormone Withdrawal versus rhTSH

**Figure 1:**
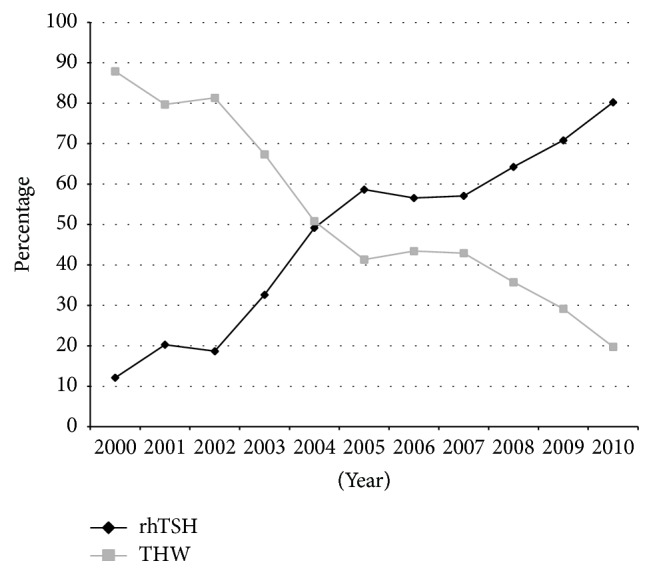
Distribution of RAI preparation (rhTSH versus THW) for patients with differentiated thyroid cancer that received radioactive iodine in the period from 2000 to 2010. Data for each year are obtained by combining all patients from all centers diagnosed in each year. rhTSH: recombinant human thyroid stimulating hormone; THW: thyroid hormone withdrawal.

Data on the protocols used to prepare for RAI ablation were available for five centers. The fraction of patients prepared with rhTSH increased from 12% in 2000 to 80% in 2010, while use of THW declined (Figure 1). There was major variation between centers, with two centers (Manitoba and Hamilton) preparing almost all patients with THW, while, in London, the majority of patients were prepared using rhTSH, and St John's and Halifax had a more even distribution of both THW and rhTSH.

## 4. Discussion

Our study indicates that, for patients diagnosed with well-differentiated thyroid cancer between 2000 and 2010, there was major variation in RAI administration for thyroid remnant ablation both across Canadian centers and over time. In addition, there was significant variation in preparation protocols and administered RAI doses between centers.

In this study, we found a high variation in fraction of patients receiving RAI between centers. For patients with lymph node involvement, the percentage of patients receiving RAI varied considerably (Table 6). For patients with negative or unknown lymph node involvement (N0 or Nx), the percentage of patients receiving RAI varied even more between centers, varying from 15 to 83% for T1, from 33 to 97% for T2, from 54 to 97% for T3, and from 75 to 100% for T4. This variation in actual RAI treatment given is consistent with the regional differences in an opinion on RAI treatment found in the cross-sectional survey by Sawka et al. [7]. Variation in management cannot be attributed to variation in the distribution of well-differentiated thyroid cancer across T stage, as found in our study, it was similar between centers and to other studies [8].

Variation in RAI treatment has also been reported in the USA by Haymart et al. [6], who found, for 2004–2008, significant regional variations in RAI use that persisted after controlling for patient and hospital characteristics. In another study, the Haymart group reported that the specialty of the primary decision maker had a major impact on use of RAI [9]. For our study, the specialty of the decision maker was not available, but most centers report this to be a multidisciplinary decision.

For patients who did receive RAI, we found significant variation in administered doses, with 1-2 centers giving an activity of 1.1–1.8 GBq in almost half of patients, while most of the other centers predominantly used an activity of 3.7 to 5.5 GBq. While the variation in RAI activity between centers has not been studied extensively, current guidelines allow for wide variation in RAI dose [5, 10].

With respect to the use of THW versus rhTSH for preparation for RAI ablation, two centers (Manitoba and Hamilton) almost exclusively used THW protocol, while the London center used rhTSH for the majority of patients. It is of interest that London and Hamilton are located in the same province (Ontario). This suggests that, for decisions regarding RAI treatment, the center for treatment is more important than the province, even though health care is a provincial responsibility in Canada,

For all centers combined, there was a major shift over the 2000–2010 decade, from predominant use of THW towards use of rhTSH for the majority of patients. A study in patients with low risk thyroid cancer found that there is no difference in ablation or recurrence rate between thyroid hormone withdrawal and rhTSH preparation [3]. Another study from the United Kingdom found that low-dose radioiodine plus thyrotropin alfa was as effective as high-dose radioiodine, with a lower rate of adverse events [4]. A recent study demonstrated reversible cognitive, motor and driving impairments in severe hypothyroidism [11]. It will be important to analyze the impact of different protocols for RAI ablation on long-term treatment outcomes, particularly recurrence rates, quality of life, and health care costs.

The use of RAI clearly increased between 2000 and 2005, while, after 2005, the fraction of patients receiving RAI decreased for T3 and even more prominently for T2 and T1 disease. These changes all occurred, even though the distribution of patients across T disease stage remained unchanged over the 10-year period, indicating this was an actual change in treatment pattern and not in patient population. In addition, most of these changes occurred before the release of the 2009 ATA guidelines. Other studies found similar results; Haymart et al. [12] reported a significant increase in the proportion of patients with WDTC receiving RAI from 40.4 to 56% between 1990 and 2008. In their report, hospital characteristics were the prime determinant of RAI treatment care pattern. This is very similar to our study in which the participating center was a major determinant of the RAI ablation rate, the preparation protocol used, and the RAI activity being administered.

Variation in thyroid cancer management has been reported in several other studies in a number of countries. A Belgium study by Van Den Bruel et al. [13], assessing the period from 2003 to 2008, indicated that regional variation in thyroid cancer incidence is associated with differences in thyroid imaging and surgery. Trocchi et al. [14] analyzed trends for thyroid cancer surgery in Germany for 2005-2006 and found that, despite an identical health care system for the whole country, there was considerable regional variation in the proportion of total thyroidectomies. Variation in treatment between countries may also be due to lack of availability of diagnostic and therapeutic modalities, and this may create barriers to implementation of thyroid cancer guidelines [15].

There are several limitations to our study. Our study database may not be representative of all thyroid cancer cases occurring in the respective regions as it was mostly accrued on a referral basis rather than that of a regional cancer registry. The pattern of referral of patients to tertiary referral centers may have changed over time. Enrollment into our study database, while being high (over 90% of patients consented to enroll), was dependent upon patients' consent. In addition, some centers started patient enrollment only in the 2005–2007 period, with some retrospective enrollment for 2000–2004, so these centers were underrepresented in the 2000–2005 period, which predominantly includes patients from London and Manitoba, the two largest contributors to our registry. Participation in the study occurs on voluntary basis. Our study does not include data from Quebec and provinces west of Manitoba. It would be interesting to know if including data from these areas would change variation between regions.

Our study also has various strengths. We include actual data from a very large group of patients and obtained actual practice information on the fraction of patients receiving RAI, specified per stage and per center. In addition, we also obtained data on the RAI activity that was actually administered and we analyze time trends for the method of preparation for RAI ablation, that is, thyroid hormone withdrawal versus use of rhTSH.

## 5. Conclusion

This study in 3072 patients from seven Canadian thyroid cancer centers found that there was, for the period of 2000–2010, major variation in use of RAI treatment between centers across Canada. In addition, there were major changes over time with the use of RAI first showing an increase up till 2005 and then gradually decreasing for all patients except T4 disease. Finally, there was a shift from predominant use of thyroid hormone withdrawal to use of rhTSH in the majority of patients. Our data show that a wide variation is present in a single-payer, government-funded system such as in Canada and that even though the provinces carry the primary responsibility for delivering health care, the variation appears to be more center than province dependent.

## Figures and Tables

**Table 1:** Characteristics of all participants (*n* = 3072).

Age (years, mean ± SD)	46.9 ± 14.7
Male/female (%)	21/79
Histology	
Papillary	2874 (94%)
Follicular	198 (6%)
TNM staging	
T1	1476 (48%)
T2	829 (27%)
T3	690 (22%)
T4	77 (3%)
Nx/N0	2452 (80%)
N1a/N1b	620 (20%)

**Table 2:** Patients with well-differentiated thyroid cancer over time (all centers combined).

Year of diagnosis	TNM stage								Number/year
	T1		T2		T3		T4		
	N1a/N1b	N0/Nx	N1a/N1b	N0/Nx	N1a/N1b	N0/Nx	N1a/N1b	N0/Nx	
2000	5 (4)	54 (39)	5 (4)	42 (30)	7 (5)	22 (16)	3 (2)	2 (1)	140
2001	10 (7)	49 (36)	5 (4)	35 (26)	12 (9)	16 (12)	7 (5)	3 (2)	137
2002	17 (10)	67 (41)	4 (3)	39 (24)	9 (6)	21 (13)	3 (2)	3 (2)	163
2003	13 (8)	63 (38)	2 (1)	49 (29)	8 (5)	28 (17)	2 (1)	3 (2)	168
2004	13 (6)	92 (44)	5 (2)	50 (24)	10 (5)	32 (15)	6 (3)	0 (0)	208
2005	18 (6)	128 (43)	8 (3)	74 (25)	19 (6)	43 (14)	7 (2)	2 (0.7)	299
2006	18 (6)	137 (43)	14 (4)	96 (30)	20 (6)	34 (11)	0 (0)	2 (0.6)	321
2007	19 (6)	148 (41)	10 (3)	79 (22)	39 (11)	61 (17)	4 (1)	3 (0.8)	363
2008	46 (10)	182 (39)	23 (5)	84 (18)	46 (10)	72 (16)	9 (2)	2 (0.4)	464
2009	18 (4)	194 (40)	23 (6)	83 (20)	34 (8)	62 (15)	6 (1)	1 (0.2)	421
2010	30 (8)	155 (40)	16 (4)	83 (21)	40 (10)	55 (14)	7 (2)	2 (0.5)	388

*P*-trend (2000–2010)	NS	NS	0.03	<0.01	<0.01	NS	NS	<0.01	

*P*-trend (2006–2010)	NS	NS	NS	0.01	NS	NS	NS	NS	

Number/stage	207	1269	115	714	244	446	54	23	3072

Data are presented as *n* (%), with the percentage referring to fraction of patients per year.

Percentage values are rounded to the nearest number, unless <1%.

Trend analysis was done to for TN stage as fraction of total number of patients per year. Only two centers (London and Winnipeg) included patients before 2006; therefore, a separate trend analysis was performed for 2006–2010.

NS: nonsignificant.

**Table 3:** Total number of patients with well-differentiated thyroid cancer by stage and center.

Center	TNM stage								Total
	T1		T2		T3		T4		
	N1a/N1b	N0/Nx	N1a/N1b	N0/Nx	N1a/N1b	N0/Nx	N1a/N1b	N0/Nx	
London	84 (5)	496 (29)	48 (29)	300 (18)	136 (8)	176 (10)	16 (0.9)	8 (0.5%)	1264
Halifax	21 (9)	144 (64)	3 (1)	31 (14)	7 (3)	17 (8)	1 (0.4)	0 (0)	224
Winnipeg	56 (6)	355 (38)	45 (5)	223 (24)	80 (9)	138 (15)	27 (3)	12 (0.3)	936
Toronto	19 (5)	167 (47)	7 (2)	102 (29)	6 (2)	49 (14)	2 (0.6)	1 (0.3)	353
St. John's	11 (7)	52 (44)	2 (1)	32 (21)	6 (4)	46 (30)	2 (1)	1 (0.7)	152
Hamilton	12 (10)	40 (44)	9 (8)	15 (13)	9 (8)	13 (11)	6 (5)	1 (0.9)	105
Fredericton	4 (11)	15 (40)	1 (3)	11 (29)	0 (0)	7 (16)	0 (0)	0 (0)	38

Data are presented as *n* (%), with the percentage referring to fraction of patients per center.

Percentage values are rounded to the nearest number, unless <1%.

There were no major differences between centers.

**Table 4:** Percentage of patients receiving RAI over time (all centers combined).

Year of diagnosis	TNM stage								Overall by year
	T1		T2		T3		T4		
	N1a/N1b	N0/Nx	N1a/N1b	N0/Nx	N1a/N1b	N0/Nx	N1a/N1b	N0/Nx	
2000	80%	43%	100%	48%	71%	59%	33%	50%	51%
2001	70%	35%	100%	60%	83%	44%	71%	100%	55%
2002	82%	49%	100%	74%	89%	57%	67%	67%	64%
2003	69%	54%	100%	78%	88%	93%	50%	67%	71%
2004	77%	57%	100%	82%	90%	91%	100%	N/A	73%
2005	83%	73%	75%	91%	89%	91%	75%	100%	82%
2006	78%	61%	93%	84%	90%	88%	N/A	50%	75%
2007	100%	52%	90%	77%	95%	82%	50%	100%	71%
2008	76%	45%	83%	71%	91%	88%	78%	100%	67%
2009	100%	36%	87%	66%	94%	84%	100%	100%	60%
2010	60%	28%	69%	61%	75%	69%	86%	100%	51%

*P*-trend (2000–2010)	NS	<0.01	0.03	NS	NS	0.045	NS	NS	0.03

*P*-trend (2006–2010)	NS	<0.01	NS	<0.01	0.03	0.04	NS	NS	<0.01

Overall by stage	79%	48%^*∗*^	86%	73%	88%	80%	76%	83%	

Data are presented as % per year, with the percentage referring to fraction of patients receiving RAI.

Trend analysis was done to compare fraction of patients receiving RAI for each TN stage over time. Only two centers (London and Winnipeg) included patients before 2006; therefore, a separate trend analysis was performed for 2006–2010.

^*∗*^ *P* < 0.01 compared to N1a/N1b group; NS: nonsignificant.

**(a):** 

Center	Guidelines	Risk category	Dose (GBq)	Population	Protocol changes 2000–2010

London,ON	ATA	Intrathyroidal disease (T1–T3, multifocal)	3.7	ATA guidelines	*Prior to 2009*: all papillary >1 cm, multifocal micropapillary with aggregated diameter ≥1 cm, all follicular, all N1 and M1
					*2009-2010*; no therapy for T1 except of aggressive variants RAI doses have not changed
		N1, ETE	5.5		
		M1	7.4		

Halifax, NS	Local guidelines adapted from ATA	Very low risk	1.1	Pts with nodal disease; P T3 and P T4; Other risk factors: lymphovascular invasion, perineural invasion, aggressive variants	*Prior to 2009*: RAI considered for all pts except pT1 N0, young (<45) F.
		Low risk	3.7		
		Intermediate risk with significant nodal disease	5.5		*After 2009*: more selective approach of pts selection for RAI administration; RAI doses have not changed
		High risk, distant mets, gross residual disease	7.4		

Winnipeg, MB	Local (CancerCare Manitoba)	Low risk	1.1	TNM (originally)	Established in 2001
		Intermediate risk	3.7	Now individualized	No changes until 2014
		High risk	5.5		
		M1	7.4		

Toronto, ON	ATA and local	Low risk	<1.1	ATA guidelines	*After 2005*: no treatment for low risk and some moderate risk lower doses 1.1–2.8 instead of 3.7–5.5
		Moderate risk	1.1–2.8		
		High risk	>2.8		

St. John's, NL	ATA	Not provided	N/A	Almost all patients are treated	No changes

Hamilton, ON	Local	Not provided		All follicular carcinomas Papillary with nodal involvement T4 stage	Doses have been decreased gradually

Fredericton, NB	Toronto, ON protocol	Not provided		All patients except those who decline or have microcarcinomas	*Prior to 2012*: All pts went to Radiation Oncology and Whole Body Scan-based follow-up; Stim Tg and US not done routinely

**(b):** 

Centers	Low iodine diet (days)	rhTSH (thyrogen)	L-T4 withdrawal	L-T4 withdrawal time (weeks)	Liothyronine	Liothyronine protocol

London, ON	12 (10 before RAI and 2 after)	Occasional cases before 2000	Used rarely (if pt unable to get rhTSH or if patient already hypothyroid on the first visit)	4	Never	N/A
		Almost everybody since 2000		If TSH is <30, wait extra week		

Halifax, NS	14	Occasional cases from 2005, more routine from 2008	Used rarely (if pt unable to get rhTSH or if patient already hypothyroid on the first visit)	4	Yes	25 mcg po BID for two weeks, start on the day of T4 withdrawal 2 weeks off

Winnipeg, MB	9 (7 before and 2 after RAI)	Rarely	Always TSH > 30 mL/L	3	Prior to 2009	4 weeks 25 mcg TID, 2 weeks off

St. John's, NL	14	Occasional cases before 2004, after used routinely	Used infrequently, over time all pts switched to rhTSH	6	Yes	2-3 weeks on 2-3 weeks off (dose not specified)

Toronto, ON	14	Since 2008	No (except in pt with metastatic/advanced disease)	3	No	N/A

Hamilton, ON	14	Since 2009	Yes TSH was not tested	4	Yes	2 weeks on 25 mcg-bid, 2 weeks off

Fredericton, NB	14	Since 2012/2013	Rarely used (only if patient is unable to access rhTSH TSH goal >35 mL/L)	2–5	No	N/A

**Table 6:** Total Number of Patients by stage and per center receiving RAI.

Center	TNM stage								Overall by center
	T1		T2		T3		T4		
	N1a/N1b	N0/Nx	N1a/N1b	N0/Nx	N1a/N1b	N0/Nx	N1a/N1b	N0/Nx	
London	81 (96%)	325 (66%)	45 (94%)	288 (96%)	134 (99%)	171 (97%)	16 (100%)	8 (89%)	1068 (84%)
Halifax	16 (76%)	86 (60%)	3 (100%)	25 (81%)	4 (57%)	12 (71%)	1 (100%)	N/A	147 (66%)
Winnipeg	29 (52%)	55 (15%)	33 (73%)	89 (40%)	57 (71%)	74 (54%)	25 (93%)	11 (92%)	373 (40%)
Toronto	14 (74%)	80 (48%)	6 (86%)	79 (77%)	6 (100%)	44 (90%)	2 (100%)	N/A	231 (65%)
St. John's	10 (91%)	43 (83%)	2 (100%)	31 (97%)	6 (100%)	44 (96%)	2 (100%)	1 (100%)	139 (91%)
Hamilton	11 (92%)	9 (23%)	9 (100%)	5 (33%)	9 (100%)	8 (62%)	5 (83%)	1 (100%)	57 (54%)
Fredericton	2 (50%)	8 (53%)	1 (100%)	8 (73%)	N/A	6 (86%)	N/A	N/A	25 (66%)

Overall by Stage	79%	48%^*∗*^	86%	74%	89%	80%	94%	91%	66%

There were no major differences between centers.

^*∗*^ *P* < 0.05 compared to N1a/N1b.
